# Progress and future prospect of *in vitro* spermatogenesis

**DOI:** 10.18632/oncotarget.19640

**Published:** 2017-07-27

**Authors:** Fahar Ibtisham, Jiang Wu, Mei Xiao, Lilong An, Zachary Banker, Aamir Nawab, Yi Zhao, Guanghui Li

**Affiliations:** ^1^ Agricultural College, Guangdong Ocean University, Zhanjiang, Guangdong, China; ^2^ Foreign Language College, Guangdong Ocean University, Zhanjiang, Guangdong, China

**Keywords:** in-vitro spermatogenesis, infertility, chemotherapy, radiotherapy

## Abstract

Infertility has become a major health issue in the world. It affects the social life of couples and of all infertility cases; approximately 40–50% is due to “male factor” infertility. Male infertility could be due to genetic factors, environment or due to gonadotoxic treatment. Developments in reproductive biotechnology have made it possible to rescue fertility and uphold biological fatherhood. In vitro production of haploid male germ cell is a powerful tool, not only for the treatment of infertility including oligozoospermic or azoospermic patient, but also for the fertility preservation in pre-pubertal boys whose gonadal function is threatened by gonadotoxic therapies. Genomic editing of *in-vitro* cultured germ cells could also potentially cure flaws in spermatogenesis due to genomic mutation. Furthermore, this ex-vivo maturation technique with genomic editing may be used to prevent paternal transmission of genomic diseases. Here, we summarize the historical progress of in vitro spermatogenesis research by using organ and cell culture techniques and the future clinical application of in vitro spermatogenesis.

## SPERMATOGONIAL BIOLOGY

**Figure 1:**
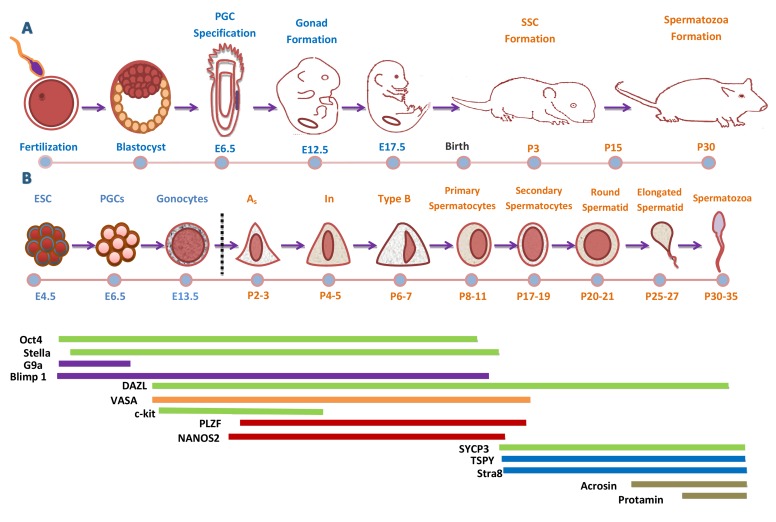
Development Cycle of Male Mouse Germ Cell **A.** Timeline of sex specification and development of male mouse. Successful fertilization promotes the formation of a zygote, which after cell divisions and cleavage will form a blastocyst. The outer layer of blastocyst gives rise to the trophectoderm while the inner cell mass (ICM) contains embryonic stem cells (ESCs). During gastrulation the blastocyst cavitates and develops the three germ layers and the epiblast. The primordial germ cells (PGCs) are specified and localize near the extra-embryonic ectoderm, at the base of the allantois. Once PGCs are specified, they migrate to the fetal gonads and undergo sex-specific development to male and female gonocytes. Subsequently, male gonocytes undergo spermatogenesis and finally produced spermatozoa at puberty (30-35days). **B.** Sequence and timeline of male mouse germ cell development and marker expression. A temporal representation of male mouse germ cells sequence and differentiation. At each germ cell developmental stage, panels of germ cell markers are expressed at definite time points to drive germline differentiation *in vivo*. At each cellular stage, important molecular and somatic signals controlling that stage are indicated above the diagram. Some of genes are observed both in human and mouse germ cells (Green Color).

Germ cells are the base of new beings and the dynamic source for genetic diversity and evolution. Although there are differences in spermatogenesis cycle duration between species, the process is similar between rodents and humans, hence, rodents are commonly used animal models to study the process of spermatogenesis (Figure 1) [1]. In mouse, progenitors of primordial germ cells (PGCs) are derived from the epiblast of blastocyst in the yolk sac in response to bone morphogenetic protein (BMP) stimulation from the nearby visceral endoderm and extraembryonic ectoderm [2]. At around the embryonic day (E) 6.0, shortly before the epiblast separates into three germ layers: ectoderm, endoderm and mesoderm, the pluripotent cells of the most proximal posterior epiblast differentiate into PGCs [3]. After that, the mPGCs start to migrate and at E 7 -8 they are observed at the base of allantois, while, at that time PGCs are referred to as migratory PGCs [4]. Then, the mPGCs are incorporated into the epithelium of hindgut, and at E 9-10 mPGCs start to migrate into the dorsal mesentery that they reach at E 10-11[5]. From the hindgut, they move to reach the gonadal ridge at E 9.5-11 and at this time they are referred to as postmigratory PGC [6]. In males mPGC differentiate to prospermatogonia at around E 13.5 and prospermatogonia located within the luminal compartment of seminiferous tubules remain arrested until after birth [7].

After birth, the prospermatogonia start to develop and the first wave of spermatogenesis begins. Around postnatal day (P) 1-2, prospermatogonia differentiate into spermatogonia, as they migrate to the periphery of the testis and become flanked by somatic Sertoli cells within the testis and peritubular myoid cells that surround the outside of the cord [8]. Although, the proteins and signaling networks involved in this transition are currently not so clear, but it has recently been shown that suppressing NOTCH signaling in Sertoli cells is important for maintaining quiescence in prospermatogonia [9].

This initial neonatal spermatogonial population is heterogeneous, undifferentiated (A_undiff_) and differentiated (A_diff_) spermatogonia are noticeable at around P 3-4. A minor fraction of the A_undiff_ spermatogonia comprises the future spermatogonial stem cell (SSC) pool, ensuring the continuous spermatogenesis during the rest of the male reproductive lifespan [10]. In mice, SSCs (A_single_) and committed progenitor spermatogonia (A_paired_ and A_aligned_) are collectively described as undifferentiated A-spermatogonia based on morphological analyses [11]. During the spermatogenesis process, the products of SSC divisions either maintain the stem cell population or produce progenitor spermatogonia for further differentiation. The differentiating product of SSCs, retain a relatively large tubular connection, termed an intercellular or cytoplasmic bridge, that results from incomplete cytokinesis [12]. These bridges are highly conserved through evolution and allow sharing of molecules between cells within a syncytium [13]. Single spermatogonia are termed A_single ,_ while, those connected by an intercellular bridge are termed as A_paired_. The commitment to enter meiosis is made with the transition of A_undiff_ into A_diff_ spermatogonia, and first differentiating spermatogonia are termed type A_1_, following, A_1_ cells divide by mitosis and form A2 cells which, in turn, divide and create A3, a division of which generates A4 spermatogonia. Next, two mitotic divisions form Intermediate and B spermatogonia [14].

The resulted B spermatogonia enter the first meiotic prophase as preleptotene spermatocytes. During differentiation, the cell-cycle duration and the quantity of germ cells decrease, such that only an estimated 39% of the expected numbers of preleptotene spermatocytes are formed [15]. Next, B spermatogonia at around P 7-10 enter the first meiosis, differentiate to primary spermatocytes and then divide to produce secondary spermatocytes [16]. The meiotic phase is quite long, ranging above a 13 day period, and at P 17-19, roughly 50% of the seminiferous tubules contain the late pachytene stage cells. [17]. Round spermatid, the earliest post-meiotic cells, are not detected till P 20-21 [18]. During the next 13 days, the round spermatids differentiate into elongating spermatids and first fertilizable sperm are seen around P35 [19].

**Figure 2:**
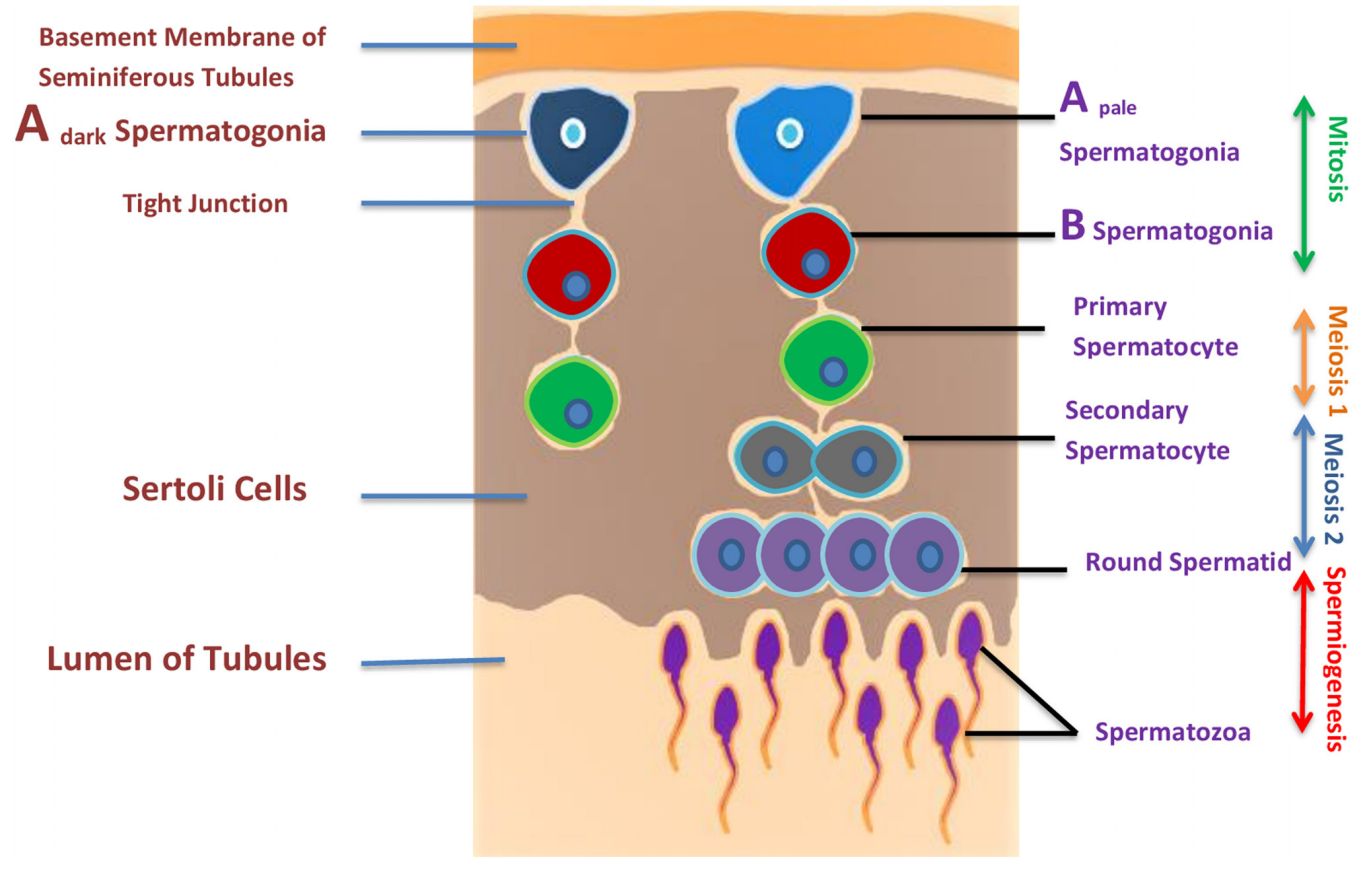
Illustration of spermatogenesis and approximate location of germ cells Spermatogenesis occurs within the seminiferous tubules of the testes of a post pubescent male. Diploid primordial germ cells (blue color) near the basal lamina of the seminiferous tubules undergo an initial mitotic division to produce diploid primary spermatocytes (green color). Primary spermatocytes migrate toward the lumen of the seminiferous tubules and begin to undergo meiosis I, resulting in haploid secondary spermatocytes (black color). These secondary spermatocytes further divide through meiosis II, producing haploid spermatids (purple color) and finally haploid spermatid produces spermatozoa by spermiogenesis.

However, little is known about the origin of human PGC, though it is believed to be similar to that in the mouse. In human, colonization of hPGC begins between 4-6 weeks of gestation [20]. Consequently, hPGCs migrate from the yolk sac endoderm through the hindgut endoderm and dorsal mesentery to the genital ridges [21]. After reaching hPGC in the testis, they are termed as gonocytes; a large nucleus and a prominent nucleolus [22]. Male sex linked genes (SRY, SOX9) start their expression in embryonic testis from about 5-6 weeks [23]. During the second trimester, mitotically active gonocytes differentiate into pro-spermatogonia [24], while, some of the pro-spermatogonia differentiate further into spermatogonia during embryonic development [25]. Then, after birth, the spermatogenic cycle initiates, which is likely the same to mice but quite a long period compared to the mouse [26] and with the exception of having only three types of spermatogonia, A_dark_, A_pale_, and B [27]. Spermatogonia A_dark_ are believed to be the reserve pool of stem cells, whereas the proliferation of active A_pale_ spermatogonia maintains spermatogenesis by balancing the production of differentiating B spermatogonia and renewing A_pale_ pool. A_dark_ cells are recognized by their dark hematoxylin staining in tissue sections as well as by other morphological criteria, while, A_pale_-spermatogonia show a less dense nuclear staining and distinctly different morphological criteria [28]. The first differentiating type B spermatogonia are visible by 4-5 year of age but only represent approximately 10% of the spermatogonial population by age 10 [29]. Studies on human spermatogonia and SSCs are very limited due to difficulty in access of human testes for research and studies purpose. Spermatogenesis occurs in successive mitotic, meiotic and spermiogenesis (Figure 2). Spermatogenesis starts in early puberty and is clinically recorded as an increased testicular volume. The meiotic process gives rise to haploid spermatocytes, which divide twice without additional DNA replication, producing round spermatids, which turn into spermatozoa, which is a morphogenic process without further proliferation [30]. The spermatozoa are released into the lumen of seminiferous tubules and are transported to the epididymis where they continue to mature. Final steps of spermatogenesis occur at puberty. In humans, whole process spermatogenesis process takes 74 days [31]. A_dark_ and A_pale_ spermatogonia are limited at the basement of seminiferous tubules. In the course of differentiation, spermatogonia move to the luminal compartment. The next layer from spermatogonia comprises meiotic spermatocytes, tailed by the post-meiotic round spermatids and lastly elongating spermatids. At the finale of this progression, morphologically-mature sperm are released into the fluid-filled lumen. (Figure 2)

The mutual interaction between germ cells and Sertoli cells is crucial for germ cell development and differentiation. The cytokines produced by Sertoli cells play an important role in spermatogonial and spermatocyte development, junctional integrity, and the function of immunoregulatory cells present in the interstitium [32]. Peritubular myoid cells surround the seminiferous tubules and express androgen receptors from fetal life to adulthood. Leydig and peritubular myoid cells are contributors of the testicular stem cell niche in mammals [33]. Follicle-stimulating hormone (FSH) and androgens (mostly testosterone) are also important for spermatogenesis. FSH is crucial for Sertoli cell population, while, androgens affect the functional completion of meiosis and postmeiotic sperm differentiation and maturation [34].

## MOLECULAR MARKERS IN SPERMATOGENESIS

Spermatogenesis is a complex developmental process and it is supported by precise and orderly regulation of gene expression (Figure .1). During mouse development, germ cell fortune is assimilated in mammalian epiblast cells and results in the formation of mPGC. The specification of mPGC involves the synergistic action of BMP members of the transforming growth factor type β superfamily [35]. These proteins bind to receptor complexes signaling to transcription factors to target specific genes. Of this family, Bmp4 and Bmp8b emitted from the extraembryonic ectoderm, are associated with PGC formation [36]. B-lymphocyte induced maturation protein 1 (*Blimp1,* also known as Prdm1*) starts expression upon the mPGC specification [37].* The tyrosine kinase receptor c-KIT and its ligand, stem cell factor (SCF) are vital for mPGC migration and proliferation [38]. Expression of fragilis is increased in the migratory PGC, inducing expression of other germ cell-specific genes such as stella and VASA [39]. Stella works as a crucial marker for mPGCs, while Dazl and Ddx4 start their expression in mPGCs from around E10.5 and last to be expressed afterward [4]. Other genes that were identified in PGCs and germ cells belong to the piwi family, miwi and mili, which regulate PGC production and spermatogenesis [40]. When mPGC have reached the genital ridges, somatic Sertoli cells and seminiferous cords surround them, and at this time mPGC are called gonocytes and enter in quiescent stage at around E 13-15 days in mice [41]. The arrival of PGCs in the genital ridge stimulates proliferation of other epithelial and mesenchyme cells to form the undifferentiated gonad composed of two compartments. The first is that of epithelial cells containing the PGCs, and the other is a stromal compartment containing fibroblasts and blood vessels. After birth, gonocytes proliferate to A spermatogonia [42]. A block in differentiation into A_1_ spermatogonia is observed in vitamin A deficient animals, demonstrating that this step is dependent on retinoic acid [43]

SSCs experience self-renewal divisions, thus upholding the stem cell population and the balance between self-renewal and differentiation is critical to sustain spermatogenesis throughout the lifespan. This small population of SSCs is responsible for the production of 10^9^ sperm per day throughout the male mouse reproductive lifespan [44]. A number of genes have been reported to intricate this balance, like PLZF [45] and NANOS2 [46]. While, Ngn3 gene is a typical gene of SSCs, and PCNA is specific gene for SSCs proliferation [47]. Other pre-meiotic markers present on SSCs include Oct4 [48], α6-integrin, GPR125, GFR-α1[49], Ty1, CD9 and β1-integrin, RET and CDH1 [50]. Spermatogonia, with the help of mitosis differentiate to A_1_ spermatogonia and at this time expressions of tyrosine kinase receptor c-KIT [51] and CYCLIN D2 [52] have been reported. The spermatogenesis and oogenesis specific helix-loop-helix 1 (SOHLH 1) proteins marker is expressed in A_1_-A_4_, Intermediate and B spermatogonia [53].

At the end of mitosis, B spermatogonia differentiate into pre-leptotene spermatocytes and the resulting germ cells enter in meiosis, a key step in spermatogenesis through which diploid germ cells divide and differentiate into haploid spermatids [54]. During the pre-leptotene stage, DNA is duplicated, followed by meiotic prophase 1 and its initiation depends on DAZL (RNA-binding protein). The presence of DAZL allows the germ cells to respond to retinoic acid that, in turn, induces expression of STRA8 [55]. The meiotic prophase 1 can be partitioned in four cytological phases: leptonema, zygonema, pachynema and diplonema. In leptotene spermatocytes expression of SYCP2 [56] and SYCP3 [57] genes have been noted, while in zygonema and pachynemant; SYCP1 expression is dominant [58]. After meiotic prophase 1, when the synaptonemal complex (SC) has been dismantled at diplonema, the next stage is metaphase 1 and the ablation of the MutL homologs MLH1 and MLH3 in mice can lead to metaphase 1 arrest [59]. During anaphase 1, the meiotic cohesin subunit REC8 is sliced off from the chromosome arms but secured at the centromeres by the protein SHUGOSHIN-2 in order to prevent premature separation of the sister chromatids [60]. Finally, at the metaphase II/anaphase II transition, the lasting REC8 molecules at the centromeres are cleaved off, thus letting separation of the sister chromatids and the ultimate generation of haploid round spermatids [61]. Protamine 1, protamine 2, and testis angiotensin-converting enzyme (t-ACE) genes are expressed in late spermatid [62]. HOP, SPAG6 and TEKSTIN-T proteins are involved in axoneme formation [63]. Cytoplasmic removal is an important process ensuring the development of compact and slender spermatozoa, while CAPZA3 gene is expressed during cytoplasmic removal.

Human PGC express genes like as POU5F1/OCT4, GAGE, MAGE-A4 and KIT [64], while Gonocytes express markers such as MAGE-A4, DAZ, KIT, PLAP, POU5F1, TFAP2C/AP-2γ and UTF1 [65]. The decision of gonocytes to enter the female or male germ cell pathway is influenced by the somatic compartment surrounding the germ cells. In males, mesenchymal, Sertoli and Leydig cells are crucial for the formation of the male gonadal paracrine and endocrine interactions [66]. The initiation of the male path of sexual development is dependent on the activation of several genes such as DHH, FGF9, M33, DMRT1, AMH, SRY and SOX9 [67]. In males, gonocytes migrate to the basal membrane and become spermatogonia while human SSC, same as rodent, express markers such as POU5F1 or TFAP2C throughout their life [68]. A multitude of genes are related to the different types of spermatogonia such as, SOX2, MYC and NANOG are related to A_dark_, CDH1, KIT and STK31 are related to A_pale,_ while, CD9, NGN3 and GFRA1 are related to type B spermatogonia in humans [69]. The pre-meiotic molecular markers in human and rodent are quite similar such as GPR125, PLZF, GFR-α1 and Ty1, but the meiotic and post-meiotic phases have different of gene markers (Table.1).

**Table 1:** Summarized difference in gene marker of human and mice during spermatogenesis

Stage	Human	Mice
Pre-meiotic	CD133, MAGE-A4, TSPY	NGN3, RET, CDH1, Stra8
Meiotic	TH2B	LDH, Crem1
Post-meiotic	TP1	Acrosin, SP-10

## *IN-VITRO* SPERMATOGENESIS TECHNIQUES

Spermatogenesis is a highly organized process of cell proliferation in seminiferous tubules and terminal differentiation, which leads to the formation of mature spermatozoa. It is a highly organized process and many types of somatic cells contribute to spermatogenesis. *In-vitro* cultures aim to emulate and abridge this subsequent environment in order to replicate the successive development of spermatogonia by the way of mitosis and meiosis for the production of haploid male germ cells. To achieve this objective, many researchers have tried different culture models and other stimulating factors in medium, as male infertility is one of the major health problems in our society. Recently, the interest in potential use of *in-vitro* spermatogenesis for the treatment of infertility has increased after successful ex vivo production of functional sperm from immature germ cells by Sato et.al [70] by using a more complex organ culture system.

The purpose of evolving the culture technique is to decrease the complexity of the spermatogenesis process into its nominal parts to study, manipulate and fully understand the connected step of this process. But there is also highly pragmatic reason to develop an effective culture technique by which haploid spermatozoa could be produced from diploid germ cells (originate from an infertile patient) with the capability to fertilize an oocyte and produce offspring. A method with the capability to produce haploid sperm would be of an advantage to both researchers and men. Lastly, an improved *in-vitro* spermatogenesis culture method would assist the *in-vitro* fertilization (IVF) treatment for patients with non-obstructive azoospermia (NOA) [71]. Thus, there is significant need of an effective method for *in-vitro* spermatogenesis. Different methods have been proposed, and these vary from organ culture system to three-dimential culture and isolated cell culture method with variations in medium.

## ORGAN CULTURE TECHNIQUE

**Figure 3:**
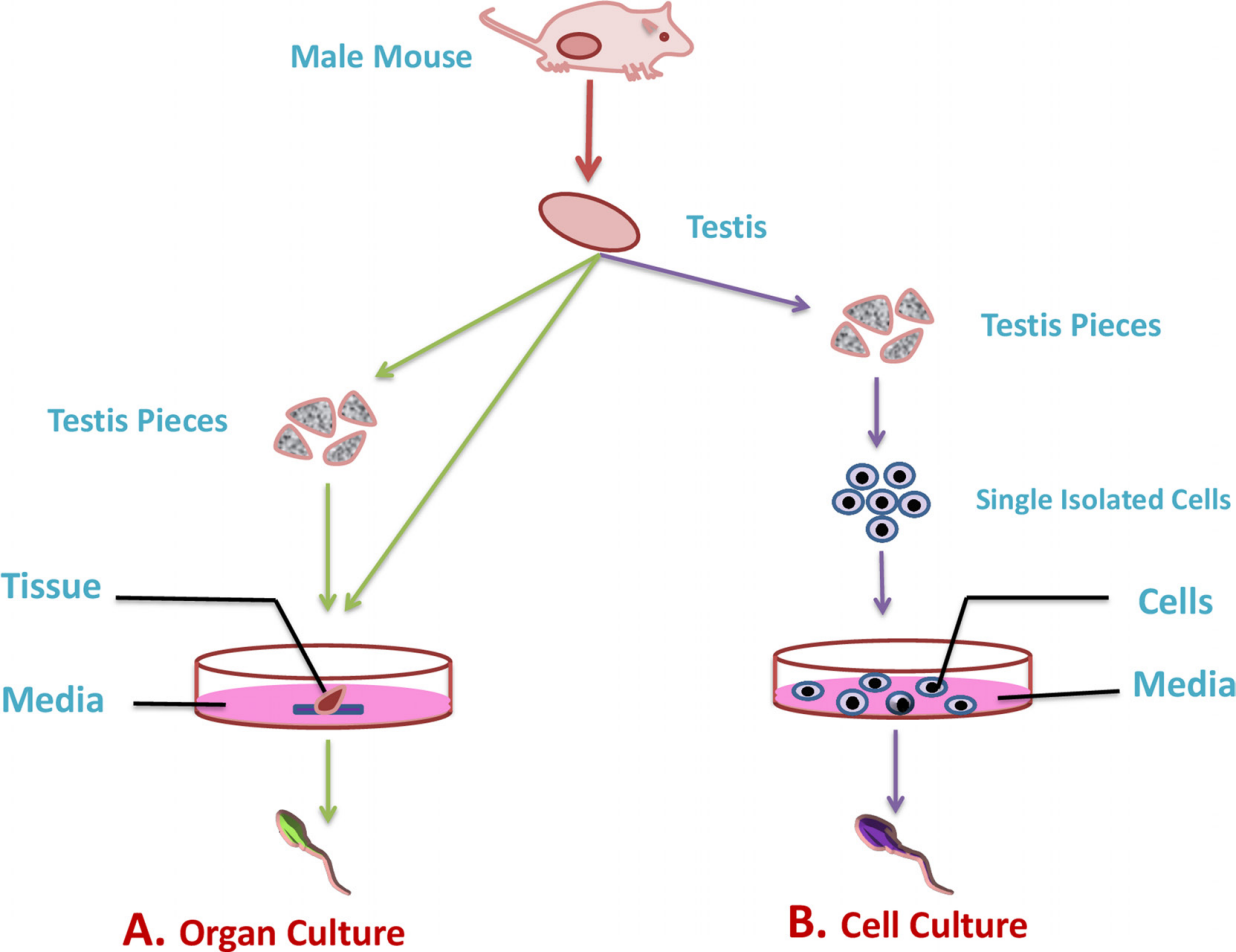
Illustration of *in-vitro* culture techniques for spermatogenesis The most commonly used technique to understand the mechanisms that lead to the differentiation of male germ cells from their spermatogonial stem cells through meiosis to give rise to mature haploid spermatozoa are organ culture and isolated cell culture technique. **A.** In organ culture technique the complete testis or its fragments are used to culture in artificial environment (green arrow). **B.** While, in cell culture technique isolated germ cell from testis are cultured in artificial environment (purple color).

A technique for maintenance or growth of animal organs *in-vitro* is known as the organ culture technique (Figure 3). *In-vitro* spermatogenesis was accomplished typically using the organ culture method with numerous ways to culture a tissue fragment or small organ until around the 1970s. In 1920, for the first time in history, *in-vitro* differentiation of germ cells was reported [72] while using the organ culture method and after that, many attempts have been made for progressive differentiation of germ cells. The core benefit of this method is that germ cells uphold their spatial arrangement and their usual cellular and micro-environmental arrangement when grown *in vitro*. Later, in 1937, progression of spermatogonia within cultured newborn mouse testes to the pachytene stage was reported [73], but the main trial in the *in-vitro* spermatogenesis process was the ability to sustain viable testicular tissue during culture. In 1959, Trowell designated a culture method in which he placed rat testis tubules in a cavity slide, while using Eagle’s minimum essential media (MEM) at 37 °C with 5 % CO2 air. The yield was viable only for 6 days. In 1964, the testis of rats at different ages were successfully cultured by adding stimulating factors including the follicle-stimulating hormone (FSH), and the human chorionic gonadotropin (hCG), while, the incubation temperature was maintained at 31°C [74]. Although modified culture conditions of rat testis were maintained for around 4 weeks, cell differentiation was not observed. Later in 1965, the same group observed the differentiation of spermatogonia to spermatocytes within 2 to 3 weeks ofculture [75] .

Matte and Sasaki (1971) observed spermatid by culturing the adult human testis tissue for 32 days in 20% FBS supplemented Media [76], but *in-vitro* progress was not confirmed. Subsequently, other scientists also confirmed the positive effects of FBS on differentiation [77]. Technological developments and advanced techniques including the electron microscope [78] and flow cytometer (1C, 2C, and 4C) [79] also solved the stage distinguishing problem of ex-vivo spermatogenesis. However, haploid cells never differentiated *in-vitro* and perished before getting to the elongated stage. To overcome this problem, the organ enzymatic digestion method was introduced later. Rat seminiferous tubules instead of the complete testis organs were firstly used for *in-vitro* spermatogenesis and more than 50% haploid cells (1C) were found after 40 days of culture [80]. In, 1998, ex-vivo production of fertile haploid cells by biopsy of azoospermic patient’s testis was also reported [81]. Maintenance of tubule cultures for more than a few days could be difficult due to high chances of tubule lumen collapse, change circulation kinetics for growth factors and metabolites. This is a valuable technique especially for IVF with NOA patients [82]. However, this system appears less efficient than healthy *in-vivo* spermatogenesis; it could be due to the improper ratio of differentiation to apoptosis. In order to find a reassuring use of *in-vitro* spermatogenesis in the future, it was vital to check if cryopreserved sections would show the same ability as fresh samples. A study was conducted on mouse cryopreserved testis and results showed that cryopreservation did not hinder progression of ex vivo spermatogenesis. Numerous organ culture studies have been done to find the ideal situations for complete spermatogenesis, although different factors and conditions have been shown to induce proliferation of male germ cells *in vitro*, but still many obstacles remained unsolved including the biological marker of SSC, self-renew of SSC and optimal conditions for *in-vitro* spermatogenesis.

**Figure 4:**
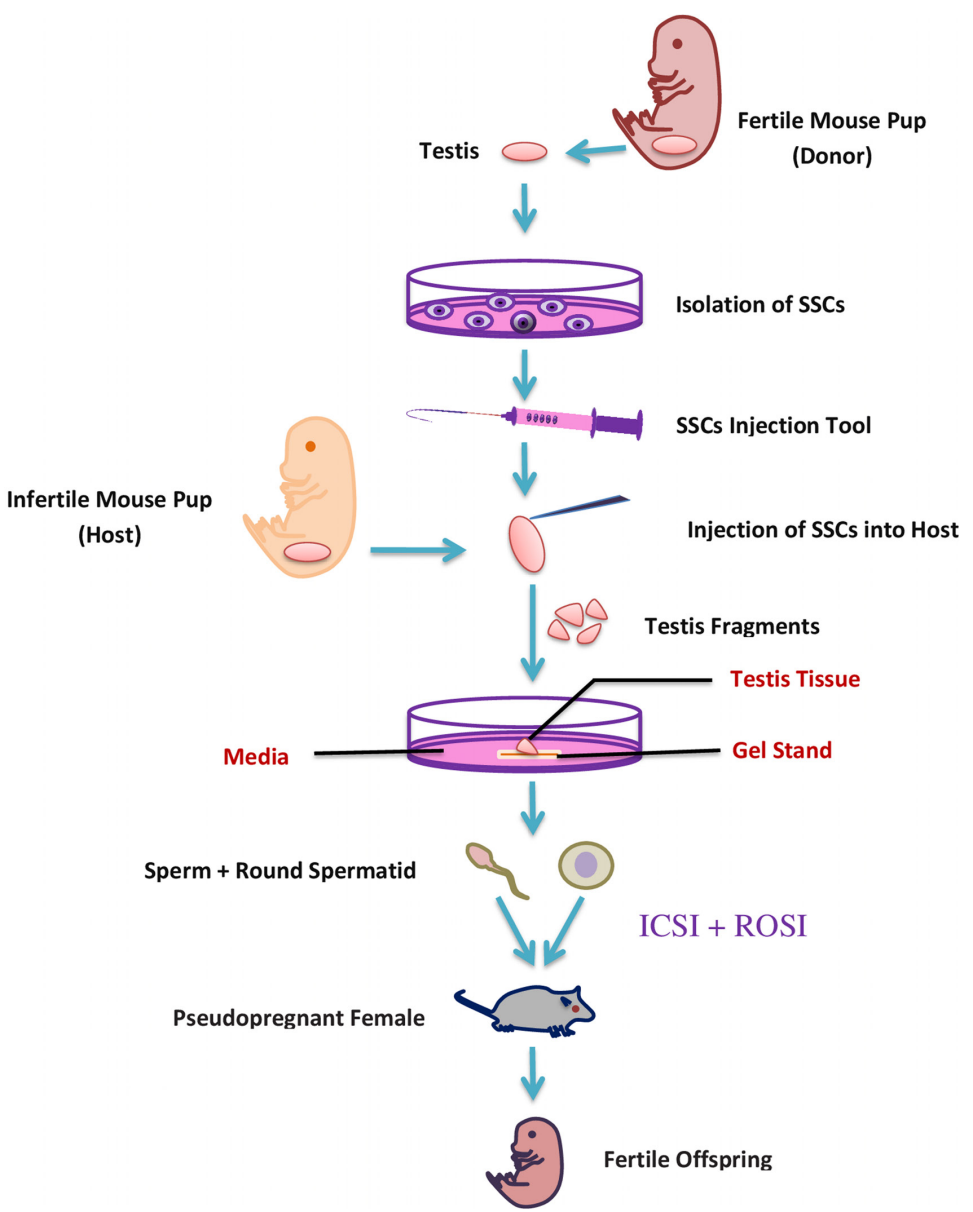
A schematic representation of *in-vitro* transplantation method

It has been proposed that fluid flow within the seminiferous tubules generates a definite confined gradient of paracrine/autocrine factors dynamically formed in basal, adluminal, and luminal compartments [83]. Within the three-dimensional (3D) tissue structure, concentration gradients might exist for any soluble culture-medium component consumed or produced by endogenous cells. Based on that theory, a new modified system of agarose gel stand has been used. Immature transgenic mouse (Acr-GFP, Gsg2 GFP) testis fragments cultured on agarose gel stand were successfully progressed to haploid spermatid [70]. The testis tissues were separated by forceps into two to eight pieces and were cultured in three different basic culture mediums (alpha-MEM, DMEM, StemPro-34 SFM) and supplemented with FBS. Sato et al. (2011) successfully produced spermatid and sperm, using the same agarose gel stand protocol. The produced spermatid and sperm fertility was checked by a round spermatid injection (ROSI) and Intracytoplasmic sperm injection technique (ICSI), respectively [84]. Additionally, the same results from cryopreserved samples instead of fresh testis showed the likely possibility for future clinical application [70]. The testes of a transgenic mouse were used to culture SSC and the following SSC were transplanted into an infertile neonatal mouse. The transplanted SSC differentiated into fertile haploid spermatids and sperm and gave rise to healthy offspring through micro-insemination [85]. *In-vitro* transplantation techniques (Figure 4) can help to diagnose the spermatogenic failure due to a micro-environmental defect in their original testes, because this method supports the differentiation of SSC to sperm. Afterward the same group also produced fertile sperm from a genetically defective infertile male like c-Kit ligand (KitL) mutation [86], and this study opened a new therapeutic strategy for patients with genetic spermatogenesis defects. Recently that group also produced fertile haploid germ cells from adult mouse testis, but the efficiency was very low compared to neonatal testes [87], so there are numerous perilous challenges which remain to be addressed in order to make the organ culture method useful in clinical application for male infertility.

## ISOLATED CELL CULTURE TECHNIQUE

In the 1980s, a number of researchers tried to reproduce spermatogenesis using dissociated testicular cells rather than organ culture (Figure 3). Cell culture using enzymatically dispersed cell suspensions provide good support of the cells *in vitro* when modern cell culture media and incubators are used. Dissociated cell culture represents a perfect method for considering the gene expression and the roles of testicular factors in differentiation.

Promoted maturating response of dissociated testicular germ cells (20- to 35-day-old rat) was observed in serum-free and hormone/growth factor-supplemented medium [88]. Subsequently, in 1989, testicular cells of a 14 day old rat were cultured on type 1 collagen gels using a medium composed of a 1:1 mixture of Ham’s F12 medium and Leibovitz’s L15 medium (F12-L15 medium) with 10% (vol/vol) FBS and the cells survived up to 14 days in culture, while haploid cells were found after 10 days of culture [89]. In the 1990s, there were two reports providing the evidence that isolated germ cells could be progressed all the way to the haploid stage [62, 63]. Subsequently, in 2002, a research group from the USA reported the production of haploid spermatid from telomerase-immortalized mouse type A spermatogonial cells in the presence of stem cell factor (SCF) [92].

A perfect culture model would include the combination of somatic and germ cells alike the spatial provisions inside the seminiferous epithelium, for the maintenance of the spermatogonial stem cell and suitable equilibrium of self-renewal and differentiation in the pre-meiotic phase of spermatogenesis. In order to improve the micro environment for *in vitro* differentiation of isolated cells, research was done in 2004, to check the effect of hormones, growth factors and feeder cells on spermatid [93]. Mouse spermatid were cultured in DMEM with FBS and supplemented with follicle stimulating hormone (FSH), testosterone and co-culture (Feeder cell). The investigator noticed the differentiation of spermatid into elongated spermatid at the 2^nd^ day of culture in the hormones supplemented group. Subsequently, based on this finding, in 2010, spermatogonia and Sertoli cells from immature (3-5 month age) buffalo testes were cultured FBS based media supplemented with FSH, testosterone and retinoic acid [94]. Spermatid-like cells with a flagellum were observed after 30 days of culture, these studies showed the critical role of FSH and testosterone in spermatogenesis.

Culture conditions had encouraging effects on *in vitro* spermatogenesis by reducing the number of apoptotic germ cells [95]. Like animals, human round spermatids in co-culture with human fibroblast as feeder cells for up to 5 days also experienced spermiogenesis [96], while spermatogonia and spermatocytes co-cultured with Sertoli cells and supplemented with testosterone and FSH differentiate into late spermatids [97]. In 2006, differentiation of type-A spermatogonia of an immature (7-day-old) rat in to spermatid was observed. When spermatogonia cells were co-cultured with Sertoli cells, however, the resulted spermatid were not fertile [98]. These studies showed that direct cell to cell contact between germ and feeder cells seems to be vital for typical spermatogenesis, but the selection of ideal feeder cells remains reliant on preferred results, and all have definite returns and drawbacks. Research by Nagano et al. was done to check the maintenance of germ cells in the presence of different feeder cells and reported that Sertoli cell accomplished poorly, while OP9 bone marrow stroma or L fibroblast cell lines showed best results for maintenance of mouse germ cells [99]. However, the production of haploid cells for fertilization in the presence of feeder cells also raises the question about whether the use of feeder cells could epigenetically impact the health of any offspring. The current study has recommended that porcine fetal Sertoli cells are proper to indorse the development of human spermatids [100].

In 2011, the use of biocompatible scaffolds was another effort to improve the efficiency of *in vitro* spermatogenesis [101]. Testicular isolated cells from immature rats were seeded on biodegradable poly scaffolds soaked in 10% FBS media and after 18 days of culture, 65% of cells were successfully attached the scaffolds with 75% viability. The differentiation rate of germ cells was also higher compared to cells seeded on a monolayer; however the fertility of produced haploid cells was not checked. Human isolated SSC were culture in a media supplemented with Knockout serum replacement (KSR) instead of FBS, and results showed that KSR promoted the differentiation [102]. In 2014, Wang et al. reported the successful generation of haploid spermatid from mouse SSC without using KSR supplementation. The isolated cells were cultured in 10% FBS supplemented media for 3 days, subsequently the cells were treated with medium enriched with retinoic acid (RA) for differentiation and after 2 days the cells were moved again into the initial media [103]. This study suggested that SSCs can be differentiated into haploid cells by simply culturing the SSCs in RA. However, the efficiency of haploid cells production was low and the fertility of haploid produced cell was also not checked. It will be fascinating to conclude whether RA in mixture with suitable culture circumstances can improve the later steps of male germ cell differentiation. In conclusion, the intensive struggles of many researchers over several years have led to the development of culture conditions for optimum *in vitro* spermatogenesis, but still much more work is needed to understand the development process and the use of differentiation stimulating factors.

## FUTURE PROSPECT

*In vitro* spermatogenesis has many clinical applications and hopefully with improvement of research findings this technique will solve the male infertility problem. Current developments in cancer treatment have saved many lives, but unfortunately, cancer therapies (chemotherapy, radiotherapy) can have a deadly effect on male germ cells, including SSC and lead to infertility. *In vitro* germ cell maturation and enrichment transfer techniques could potentially help to preserve fertility, especially in pubertal males without mature germ cells. In addition, this technique could also be potentially used for the treatment and the maintenance of biological paternity of oligozoospermic or azoospermic patients.

## FERTILITY PRESERVATION OF PRE-PUBERTAL CANCER PATIENT

Due to current developments in medical treatments for cancer; control and cure of this life threatening disease has become possible for young cancer patients. Cancer can be cured by chemo- or radiotherapy, but, lifesaving treatments carry a significant risk for infertility. Consequently, with the treatment of disease, controlling the side effects of gonadotoxic therapies has also become a point of interest for young cancer patients [104].

Because chemotherapy can also negatively affect spermatogenesis [105], semen preservation is an option to reserve the chances to have their own biological children in the future [106]. Cryopreservation is accepted as a safe and useful method of preserving fertility potential in male cancer patients regardless of semen quality [107]. Although, this technique for fertility preservation is easy safe and an easily accessible method for male patients facing cancer treatment, but it is the only possibility for post-pubertal male patients, not for pre-pubertal patients.

Fertility potential of pre-pubertal male cancer patients (ongoing gonadotoxic therapies) can be preserved by two different methods; (i) minimizing the testicular damage or protect the SSC *in-vivo*, (ii) cryopreserve the testicular tissue. Deadly effects of gonadotoxic therapies can be minimized by using cytoprotective drugs, but to date no effective gonadoprotective drugs are available for use in humans [108]. Consequently, the best alternate method to preserve the fertility is the cryopreservation of testicular tissue. Pubertal testis tissue contains SSC, which can be cryopreserved as a cell suspension [109] or in tissue form [110]. Cell suspension is prepared by enzymatic digestion of testicular tissue, post-thaw viability of SSC was 29-82% in animals [111], while in humans, it was about 60 percent [112]. There are different methods to acquire sperm from samples including SSC transplantation, testis tissue grafting, and *in-vitro* spermatogenesis.

**Figure 5:**
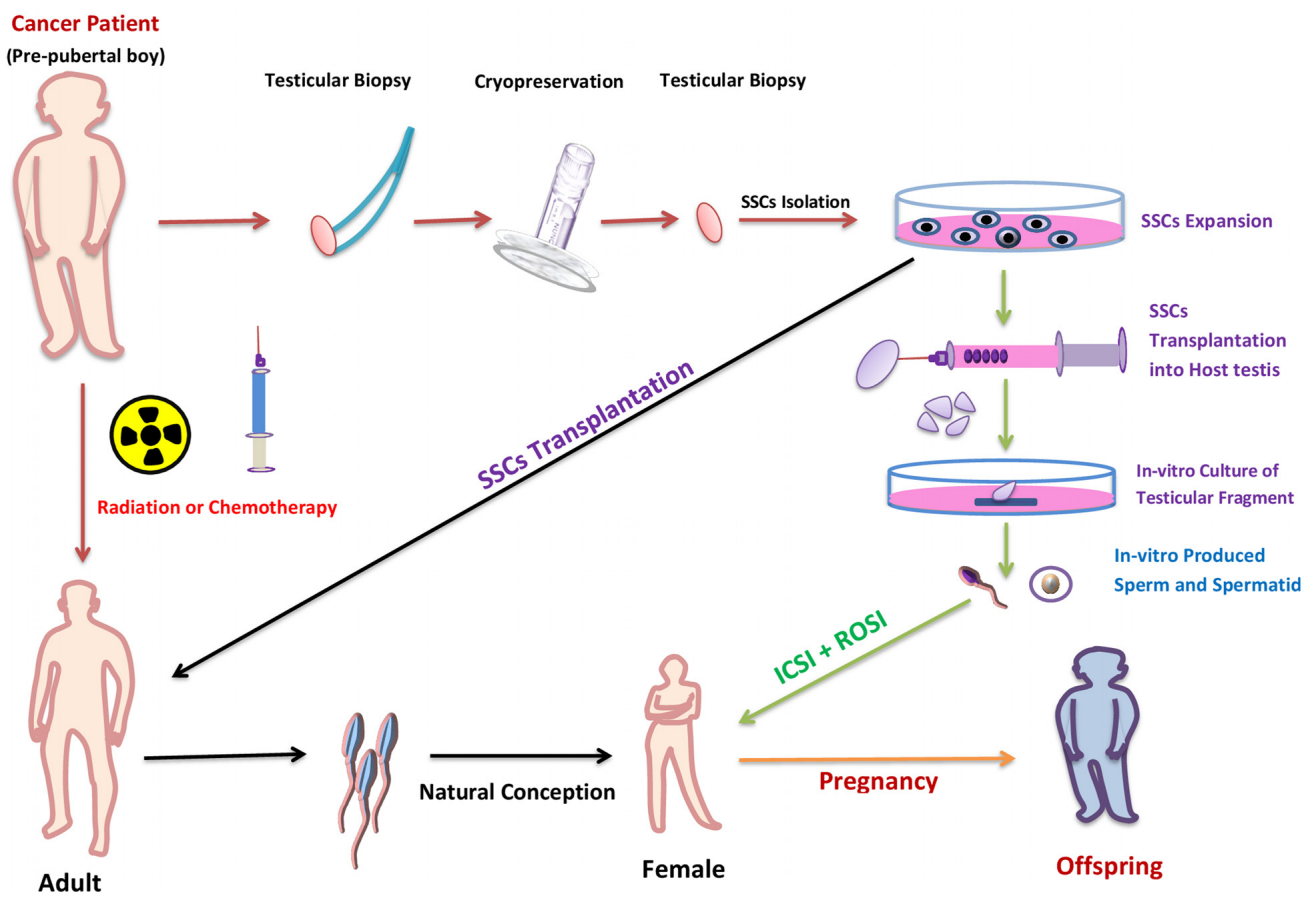
Potential use of *in vitro* spermatogenesis for fertility preservation of pubertal cancer patient In pubertal cancer patients, testis biopsy could be taken and cryopreserved before treatment with chemotherapy or radiation. After successful treatment, SSCs (isolated from frozen-thawed biopsy) would be *in vivo* auto-transplanted to the patient’s testes to restore fertility (black arrow). If, however, the patient has a risk of malignant-cell contamination in the testis; the malignant cells could be isolated by flow cytometer or differential plating before transplantation. If, after treatment the testis don’t support the spermatogenesis then the *in-vitro* isolated SSCs from frozen-thawed biopsy could be transplanted in to testicular explant from another individual (green arrows). Following *in vitro* culture of the explants, the mature germ cells would be isolated and used to fertilize human eggs by ICSI or ROSI.

In the SSC transplantation approach, the isolated cells are transplanted into testis of patients after recovery from their disease. The transplanted SSCs are renowned by Sertoli cells and move from the lumen onto the basal compartment of seminiferous tubules. The technique of SSC transplantation has been broadly researched in lab animals including mice and rats as host animals [113] and successful production of functional sperm was also reported after transplantation [114]. Like animal models, in humans the transplantation of SSC was investigated, and it was reported that 55% of the total tubular lumen of testis contained transplanted stained SSC [115]. The efficiency of SSC transplantation method depends on the quality and quantity of SSC transplanted to seminiferous tubules [116]. Consequently, to improve the success rate, increasing the number of SSC prior to transplantation is essential. The number of SSC can be increased by isolating the pure stem cell and then *in vitro* expansion of SSC and resulting SSC can be transplanted in seminiferous tubules for further development and to stain the spermatogenesis (Figure 5).

Cryopreservation of immature testicular tissue is increasingly applied to preserve SSCs. The efficiency of testis tissue cryopreservation by slow-freezing or vitrification has been demonstrated in several animal species, including mice, pigs, and Japanese quail yielding healthy offspring after transplantation [117]. Thus, frozen SSCs obtained via testicular biopsy prior to gonadotoxic therapy is the only best possible way to preserve the fertility in pre-pubertal patient. The first successful SSCs transplantation was reported in 1994 and resulted in fertility restoration in sterile recipient mice [118]. This technique also have been successfully applied in many other animals including pig [119], bulls [120] and primates [121]. The breakthrough was achieved when donor-derived sperm was generated from autologous and allogeneic transplantation of SSCs from rhesus monkey [114]. Subsequently, ICSI was conducted to fertilize oocytes, and embryos with donor paternal origin were finally produced. This demonstration in primates provides prospects for future clinical translation of SSCs transplantation. It should be emphasized that freezing testicular SSCs from patients with oncological disease has two advantages compared with the cryopreservation of spermatozoa, (a) SSC are easy to expand *in-vitro*, and later the SSCs can be either transferred back to the patients’ testes or cultured *in vitro* to produce haploid germ cells. Spermatozoa can’t undergo mitosis as they are ﬁnally differentiated haploid cells, thus freezing any spermatozoal population represents maintaining *in vitro* a limited part of the genotype of the patient; in contrast, SSCs are easy to expand, so, a larger amount of genetic information of the patient can be maintained.

However, the *in vitro* propagation of human SSCs is not yet feasible [122]. The other possible way to sustain the fertility is by the way of testis tissue grafting. As for animals like mice, rabbits, sheep, and pigs, testicular grafting techniques have been used and successful production of functional sperm was also reported [123]. However, to date still have no successful reports of human testis tissue grafting experiments and the efficacy of this method is also still questionable.

Fertility preservation is a vital problem for a good social life of pre-pubertal boys experiencing gonadotoxic cancer therapy because due to sexual immaturity, sperm cryopreservation is not probable. The SSC transplantation and testicular fragment grafting could facilitate the sperm production from cryopreserved testicular biopsy, but these approaches have some serious flaws, like the possibilities of presence of malignant cells in the sample. Such problems can again lead to re-introduction of malignant cells into the patient. This situation could be overcome by cell-sorting, but unfortunately it is also not reliable [124].

*In-vitro* spermatogenesis technique has minimized the possibility of cancer cell reintroduction and made this procedure possibly very advantageous in cancer patients. However, the crucial goal of producing functional sperm *in vitro* was not accomplished until the report from Ogawa group in 2010 [125], but unfortunately, they were not able to differentiate the germ cell past to the round spermatid stage. A new era in the field of regenerative medicine and a new hope for cancer patients to preserve fertility started with the report of successful haploid spermatid and sperm production, by the same group in 2011 [70]. The *in-vitro* produced elongated spermatid and sperm fertility was checked by ICSI; a method in which elongated spermatids or sperm are injected into mature oocytes, trailed by *in vitro* culture of the fertilized oocytes to form two-cell embryos, and finally the transfer of the embryos into the oviducts of pseudopregnant females [126]. Females implanted with embryos generated by *in vitro* produced haploid germ cells; successfully produced offspring, even the haploid spermatid produced in their culture condition also produced healthy offsprings by ROSI [70]. Ogawa group also checked the efficiency of the cryopreserved testis sample for spermatogenesis, and they found some of the germ cells from cryopreserved samples differentiate into elongated spermatid in their culture system [127]. The offspring produced by *in vitro* mature spermatid and sperm grew healthily and produced the next generation by natural mating. This approach, cryopreserving testis tissue tailed by *in vitro* spermatogenesis, minimized the other possible problem including the intrusiveness of the technique to patients and the possibility of re-implanting cancer cells. This technique has been reported in mouse testis tissue, but with improving the culture condition, this technique may become the way to shield and retain the reproductive capability of pre pubertal male cancer patients.

## INFERTILITY TREATMENT

**Figure 6:**
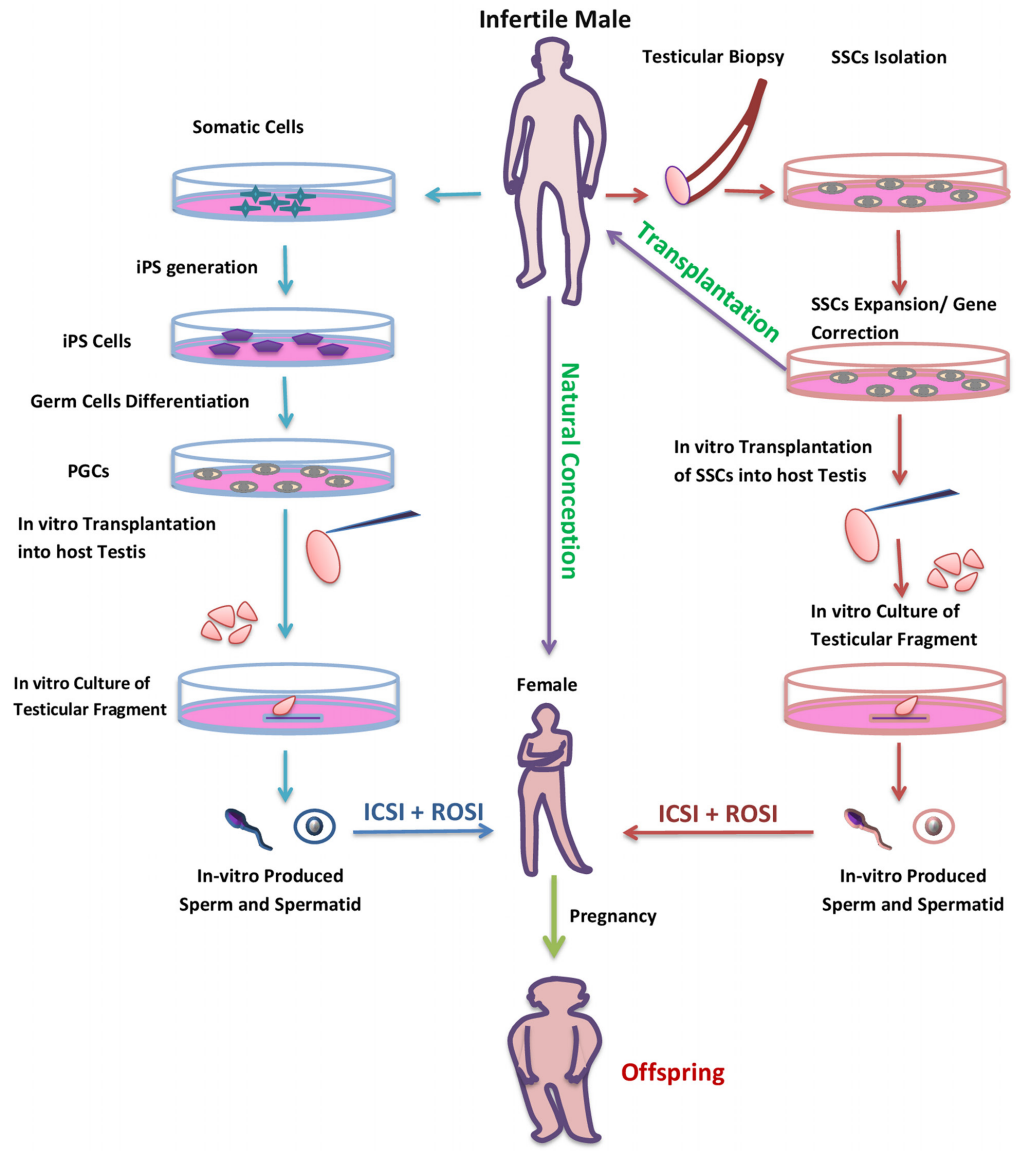
Potential use of *in-vitro* spermatogenesis for the treatment of infertility Infertile patient due to genetic defect or with a problem of low number of germ cells could be cured with *in-vitro* spermatogenesis. SSCs could be collected from such patients by testicular biopsy and following SSCs could be expand or genetic defect could be corrected by gene theory. Then, the gene corrected and expanded SSCs could be transplant in to a patient’s testis to restore fertility (purple arrow). But, for patients with Klinefelter syndrome, whose testes might have atrophied by adulthood, the only option would be *in-vitro* differentiation of SSCs to spermatozoa (red arrow). The patient without any germ cell in their testis could also have their own biological offspring by in vitro production of haploid germ cell from somatic cells (blue arrow).

Male infertility is one critical problem in our society affecting almost 7% of the total male population [128]. Infertility in males can be due to a number of reasons including endocrine problems, obstructive syndromes, and hypogonadism, but many causes are treatable (25-75%) when advanced techniques are properly applied. Recently, numerous practical advancements have reliably enhanced the chances of attaining a healthy offspring for men with impaired fertility (Figure 6).For instance, microsurgical extraction of sperm (m-TESE) in cases of low quantity and followed by the use of ICSI to attain pregnancy. Animals in which ICSI has produced normal offspring include many species and success rates in mice have been high compared to other species. The high success rate of mouse ICSI demonstrates that spermatozoa nuclei are genomically intact. In addition, ROSI also enabled azoospermia patients with presence of round spermatid in their testis, to attain a healthy offspring [129]. Survival efficiency of oocytes after ROSI is higher than after ICSI [130], it could be due to smaller size of the injection pipette for ROSI, which helps to minimize the damage to recipient oocytes. Preactivation of oocytes is necessary in case of round spermatid microinsemination because these immature gametes have no oocyte-activating capacity [131]. Although, ROSI-generated embryos develop to a two cell stage at a normal rate, but, developmental efficiency rate after embryo transfer is significantly lower in embryo generated by immature germ cells compared with mature [130]. This indicates that ROSI-generated embryos have some specific difficulties in postimplantation development compared with ICSI. Such findings suggest that male germ cells might acquire a definite capacity for supporting embryonic development at some time during nuclear condensation in spermiogenesis. Further information should be important for more efficient and safer immature germ cell microinsemination in humans as well as in other animals.

However, these techniques are useless in the case of patients having no sperm and round spermatid in their testis [132, 133]. Such patients are about 25 to 50% of total andrological cases [80, 81]. This type of aspermatogenic patient can be divided in three groups based on histopathologic findings in biopsies, including a) complete absence of germ cell [134] b) mitotic arrest during spermatogenesis [135] and c) pre-meiotic arrest [136]. Spermatogenic arrest is one of the major causes of male factor infertility. Arrest represents a situation in which spermatogenesis stops at a specific stage of germ cell development. Arrest occurs often at the spermatocytes or spermatogonial level, but most frequently arrests in the primary spermatocyte stage [137]. Interestingly, in arrest at the level of meiotic cells, these cells can be injected into oocytes to produce viable embryos and even offspring [138]. The micro-fertilization technique using primary or secondary spermatocytes and maturing oocytes may provide a possible opportunity for treating most infertile patients with spermatogenic arrest, but this experimental option has low efficiency. For instance, only 15% of mouse oocytes injected with secondary spermatocytes generated offspring [138], whereas 9% of those injected with primary spermatocytes generated offspring [139]. The use of intra-cytoplasmic spermatocyte injection technique also has been reported in humans with offspring-deriving efficiency of approximately 3% [140]. According to the authors, technical difﬁculties, incomplete genomic imprinting and/or incomplete DNA repair might be responsible for the poor zygotic development. For these reasons, the use of spermatocytes to compensate for a lack of more mature male germ cells will require further experimentation. The production of offspring from oocytes injected with primary and secondary spermatocytes indicate that (a) the DNA ploidy associated problem by using such immature germ cells that have not yet completed the second meiotic division could be resolved by the use of this technique and (b) the both male meiotic division can be completed within the cytoplasm of female gametes, so, the female gamete can serve as a biochemical ‘medium’ for differentiation of immature germ cells to mature fertile germ cells. Thus, this technique could have great clinical importance for the therapeutic management of men with complete early maturation arrest. Presently development in regenerative medicine, like SSC transplantation, xenologous systems and *in vitro* spermatogenesis could pass confidence to men with severe cases of aspermatogenesis.

**Figure 7:**
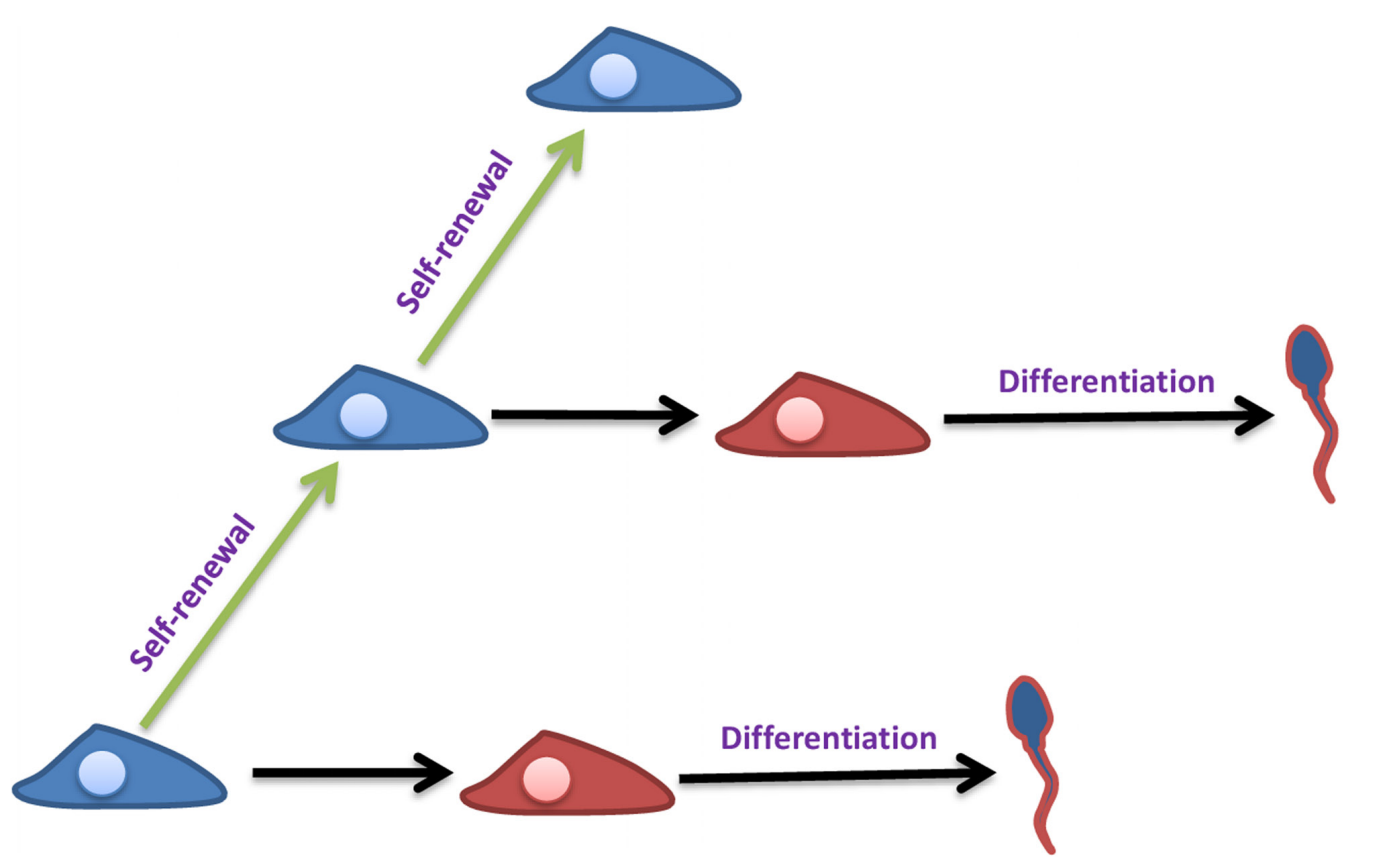
Self-renewal and differentiation of SSCs Self-renewal ability of SSCs (blue color) allows the spermatogenesis to be continuous during a male’s reproductive lifespan. They can also commit to differentiation (red color) and produce sperm.

Fertility is the continuous production of sperm through spermatogenesis process [141], while this process is maintained by SSC, self-renew cells (Figure 7) and found at the basement of seminiferous tubules [142]. Since, the report of the possibility of SSC isolation and transplantation into testis [118]; this method has been explored to cure the problem of infertility [143]. SSCs are a probable tool to cure male infertility due to their capability of developing into spermatid or sperm after *in-vivo* transplantation [144]. This advanced technique of *in vitro* SSC culture and following transplantation of *in vivo* differentiation is a useful technique only if the infertile patients have no germ cells in their testis but can support spermatogenesis. If the patient testes do not support the spermatogenesis then *in-vitro* maturation of SSCs is the last possible opportunity for treating most infertile patients (Figure 6).

The technique of germ cell transplantation could also be applied across species (xenogeneic transplantation). Rat testis cells that were microinjected into the testes of immunodeficient mice developed and completed spermatogenesis [145]. Rat sperm were identiﬁed in the epididymides of recipient mice by means of their characteristic head shape. But, limitation of xenogeneic testicular stem cells was observed in less closely related species. When hamster germ cells were injected into immunodeficient mice testes they successfully colonized the tubules and completed spermatogenesis [146]. Although, hamster spermatozoa were found in recipient mice, abnormalities were noted in the shape and spermatozoa were generally lacked acrosomes. Furthermore, xenogeneic transplantation from rabbit and dog into genetically immunodeficient mice testes did not produce spermatozoa, although the cells successfully translocated to the basal compartment of the recipient [147]. Spermatogonia from less closely related species have the ability to repopulate the testis but will not differentiate, which currently renders the use of xenogeneic transplantation.

As an alternative to SSC transplantation into testicular environments, xenotransplanting technique was developed and was first successfully tried with mouse testis tissue (donor) grafted into mouse skin (recipient) [148]. This technique also has been successful applied to many donor species including dogs, cats, hamsters, rabbits, sheep, goats, horses, cattle, alpacas and monkey [149]. In the absence of alternative strategies to generate sperm *in vitro*, grafting provides an approach to ex-vivo generation of mature sperm and xenografting of neonatal and pre-pubertal testicular tissue is better compared to developed tissue as immature testicular tissue has a high regenerative capacity. Thus, Grafting is also a possible clinically applicable strategy for fertility preservation in immature patients.

*In vitro* production of germ cells from induced pluripotent stem cells (iPS) or embryonic stem cells (ES) has opened up a new possibility for infertility treatment (Figure 6) [90, 91, 92]. The ES and iPS cells generated from the individual could be *in-vitro* differentiated into male germ cell lineage [150–152], and those male germ cells for further differentiation into haploid germ cells [153], can be transplanted into testicular explant of another individual by the approach designated by Ogawa and colleagues [70]. The resultant haploid male germ cell could be isolated and used in production of healthy offspring by modern techniques like ROSI or ICSI (Figure 6). In 2006, one laboratory reported the production of mouse offspring from *in vitro* sperm derived by ES cells [154]. They established the SSC lines from ES cells following SSC underwent meiosis and produced haploid male gametes *in vitro*. The following haploid cells were used for ICSI for offspring production, however, the efficiency of offspring production was low and produced offspring died prematurely. This technique could be the worth attempting to approach a cure human infertility problems, although haploid male germ cells have been produced in humans [155], but still the functionality of produced germ cells has never been tested.

Finally, infertility due to some known gene mutation could also be cured with *in vitro* spermatogenesis by gene therapy of male germ cell progenitors *in vitro* (Figure 6). Following germ cell transplantation in patient testis for further *in vivo*-differentiation or by *in vitro* development in explant testis for production of haploid functional germ cells. In summary, all of the outcomes untaken are genuine biotechnological developments with likely potential in cases of an aspermatogenic patient. However, remarkable potential of these methods for *in vitro* production of gametes by culture techniques, much research remains to be conducted in this area.

## CONCLUSION

Significant progresses have been made during the past few years in our understanding of male germline stem-cell biology. This understanding, as well as the transplantation and ex-vivo haploid germ cells, holds great promise in treating male infertility. Providing young people undergoing gonadotoxic treatment with adequate fertility preservation strategies is a challenging area of reproductive medicine and *in vitro* spermatogenesis offers the prospect of several realistic applications. Although these techniques have only been applied in lab animals, there is reproductive technology advancement hope for the near future that these methods will also give surprising results in humans.
